# Editorial: Proceedings of Olivebioteq 2018 – Olive Management, Biotechnology and Authenticity of Olive Products

**DOI:** 10.3389/fpls.2020.00860

**Published:** 2020-07-02

**Authors:** José Enrique Fernández, Antonio Díaz-Espejo, José Manuel Martínez-Rivas, Wenceslao Moreda

**Affiliations:** ^1^Institute of Natural Resources and Agrobiology of Seville (CSIC), Seville, Spain; ^2^Instituto de la Grasa (CSIC), Seville, Spain

**Keywords:** biotechnology, abiotic stress, crop management, oil quality, table olive

OLIVEBIOTEQ'18 was the 6th International Conference on the Olive Tree and Olive Products. Held in Seville, Spain, in October 2018, it gathered 218 registered participants from 16 countries with the objective of bringing together the latest advances and knowledge in the areas of breeding and propagation; reproductive and molecular biology, genomics, and biotechnology; crop response to biotic and abiotic stresses and crop management; economics of the olive crop and olive products; table olive and olive oil quality, authenticity, technology, and by-products; and nutrition and health. For all these areas, we considered research, technological, industrial, and commercial aspects.

The publication of this special issue was one of the activities organized within the frame of OLIVEBIOTEQ'18. We contacted 252 potential contributors and received 50 abstracts. Eventually, 31 manuscripts were submitted and 21 accepted for publication. Seventeen are published in Frontiers in Plant Science, within the Crop and Product Physiology section; two in Frontiers in Nutrition, Food Chemistry section; one in Frontiers in Sustainable Food Systems, Crop Biology and Sustainability section; and one in Frontiers in Bioengineering and Biotechnology, Industrial Biotechnology section.

Three of the accepted manuscripts are related to breeding and propagation. The work by Miazzi et al. addresses the recovery and conservation of ancient and rare olive germplasm from the Apulian region (Southeast Italy). A total of 177 genotypes were recovered, analyzed, propagated and transferred to an *ex-situ* field. The identification of genotypes useful for breeding programs was key among the outputs of this work. French genotypes were studied by Khadari et al., who used molecular characterization to both elucidate their origin and facilitate cultivar management. They found high genetic diversity in the 113 studied olive accessions, suggesting that French olive germplasm resulted from the diffusion of material from Spain and Italy. With an interest in selecting cultivars with an improved ability to better adapt to new management systems, de Castro et al. developed a UAV-based high-throughput system for the improvement of olive breeding programs. The system includes a new, high-performance object-based image analysis algorithm, suitable for the quantification of tree architectural traits, especially tree height and crown area. These three approaches illustrate the key role that molecular biology and remote imagery techniques play in the identification of genotypes for breeding programs and in the identification of links between genotype and phenotype.

The role of genomics and biotechnology in plant protection is outlined in the work by Narváez, Martín et al., wherein they analyse the response of transgenic olive plants to *Verticillium dahliae* and *Rosellinia necatrix*. These plants constitutively expressed the *NPR1* gene from *Arabidopsis thaliana* as a response to the infection by both species of fungi. An embryogenic line from a seed of cv. Picual was used to obtain several transgenic plants showing different responses to a variety of pathophyte species. In another work, Narváez, Prieto et al. perform a pioneering analysis of the regeneration capacity, via somatic embryogenesis, of four wild olive genotypes with different responses to defoliation caused by *V. dahliae*. Another example of genomics, this time applied to the fatty acid composition of olive oil, is given by Salimonti et al. This group worked with the oleate desaturase enzyme encoding-gene (*FAD2-2*), which is the main gene responsible for the linoleic acid content in the olive oil. More precisely, they performed an *in silico* and structural analysis of the 5′UTR intron of the *FAD2-2* gene to explore the natural sequence variability and its role in the regulation of gene expression. Their findings reveal new structural variants within the *FAD2-*2 gene in olive, putatively involved in the regulation mechanism of gene expression associated with oleic and linoleic acid content variation. These lines of research highlight the utility of molecular approaches for the improvement of olive biotic stress resistance and oil quality.

In the field of reproductive biology, the work by Jimenez-Quesada et al. addresses the production of Reactive Oxygen Species (ROS) in the olive reproductive tissues as a response to intense metabolism. After localizing the Rboh-type gene (*OeRbohH*) through pollen ontogeny and pollen tune elongation analysis, they found that a balanced activity of tip-located *OeRbohH* during pollen tube growth is important for normal pollen physiology. With respect to fruit development, Diarte et al. studied fruit skin properties in the fruits of nine olive cultivars. They isolated cuticular membranes to analyse the composition of cuticular waxes and cutin monomers, and performed microscopy observations of fruit pericarp sections. Skin surface topography was also analyzed by means of fringe projection and significant differences among cultivars were found and discussed.

Furthermore, Muzzalupo et al. discuss an innovative plant protection approach. They investigated the *in vitro* antifungal activity of two olive extracts against a *Fusarium proliferatum* strain that causes diseases to many economically important plants. Olive extracts are known to exert anti-inflammatory, antioxidant and antimicrobial activities. Among other important findings, the authors show enhanced antifungal effects obtained with the encapsulation of leaf extracts in chitosantripolyphosphate nanoparticles before application. This result suggests that new application techniques can be developed to reduce the dosage of fungicides, which are potentially harmful to human health. Herrera et al. focused on the potential of *Dittrichia viscosa*—a plant common in olive growing areas—for the development of pest management systems in olive orchards. They studied the arthropofauna associated with *D. viscosa* and found that the plant's phenology influences the populations of a variety of arthropods, and that plants of *D. viscosa*, grown along the borders of and inside olive groves, serve as natural reservoirs of predators and *Hymenoptera* parasitoids, protecting olive trees from attacks by any *D. viscosa*-related phytophages. The work by Campos et al. describes a new quantitative PCR analysis based method for the detection of several viruses in olive trees. This innovative detection system was tested in trees of different cultivars, resulting in sensitive and reliable estimation of virus accumulation in infested trees. The system, therefore, can be an effective tool for sanitary certification of olive propagative material.

With the goal of elucidating the effect of abiotic stress on olive growing, Conde-Innamorato et al. investigated the performance of six Mediterranean olive cultivars for the temperate humid climate conditions of Uruguay. Main aspects of phenology, growth, production and oil quality were addressed. This extended study in which 10 growing seasons were considered, revealed that, although the performance of some of the tested cultivars was promising, the alternate bearing is a limiting factor. Their findings reveal a need for further research on cultivar × environment × management interactions. In an assessment of the growing conditions in the neighboring country of Argentina, Miserere et al. evaluated the response of olive trees to sap flow, stomatal conductance, and xylem anatomy to elevated temperature, as well as the effect of crop load on that response. The authors were concerned about the possible effects of climate change in that part of the world, where temperatures are already above those considered as optimal for olive growing. Once again, findings from this work outline the importance of considering the interactive effects of multiple factors when assessing olive performance. The problem of salinity is addressed by Regni et al. They induced saline stress in potted plants of four olive cultivars and studied the effect of salt stress on plant growth and the main physiological and biochemical variables. Photosynthesis, chlorophyll content in the leaves, and plant growth were negatively affected by increasing salinity, while the GSH and CAT enzymatic activities increased. In addition, proline content in leaf tissues decreased, which, in turn, altered osmotic regulation. The greater activity of the two antioxidant enzymes was effective in counteracting the effects of saline stress. The effect of water stress and its influence on the hydraulic performance of olive plants was addressed by Hernandez-Santana et al. More specifically, they evaluated whether hydraulic traits contributed to differences among water treatments in the leaf gas exchange and plant growth of four wild olive genotypes. They found that both the leaf area:sapwood area and the leaf area:root area ratios decreased with water stress, with differences among genotypes. Their findings show that hydraulic allometry adjustments at the plant level were coordinated with the physiological response in leaves, and the authors go on to outline the relevance of plant hydraulic traits, i.e., the efficiency of water transport throughout the plant's hydraulic system, as useful to both anticipate the impact of climate change and improve crop water productivity.

On irrigation, Romero-Trigueros et al. evaluated the impact of using reclaimed saline water and deficit strategies on the ripening indexes, olive yield, and oil quality of “Arbosana” trees. The combined effect of the salt and water stresses did not decrease fruit yield, but did reduce oil yield because the oil content per fruit dry weight was affected as compared to control trees. Full irrigation with reclaimed saline water decreased oil quality, but with deficit irrigation, the total polyphenols increased. These findings will be of interest to many because the use of low quality water for irrigating olive orchards is expected to increase. On fertilization, Fernández-Escobar offers a review on the question of whether the olive tolerance or resistance to biotic or abiotic stresses can be affected by the nutritional status. It seems that an adequate nutritional status improves the plant's behavior under stress conditions, and the role that certain nutrients have on a number of physiological mechanisms has already been clarified, e.g., the role of potassium on stomatal control. In this work the author explores several of these cases and focuses on the formation of a physical barrier by silicon deposition in the epidermal cells of the leaves, and on the benefits of such barrier to control pest and diseases in olive.

The work by Dias et al. focuses on the effect of pruning on both olive yield and the performance of the Side-Row Continuous Canopy Shaking Harvester. The test was performed in a high density “Picual” orchard, under different pruning treatments. Neither the average yield per tree, for the 4-year testing period, nor the average olive removal efficiency, were affected by the pruning treatment. Goldental-Cohen et al. addressed the mechanical harvesting of table olives. They evaluated the sensitivity of 106 olive cultivars to browning caused by mechanical injury, and identified 14 resistant genotypes that could serve as table olive cultivars. Their findings suggest that cuticle thickness can be an indicator to identify table olive cultivars suitable for mechanical harvesting.

In relation to olive oil quality, 11 olive cultivars were evaluated by El Riachy et al. Their findings show that local and foreign varieties growing in Lebanon produce good quality olive oil. They identified the time course of principal quality variables including ripening, and concluded that further investigations for the characterization and authentication of Lebanese olive oil are required. Concerning olive oil technology, Veneziani et al. studied the impact of the pulsed electric field on the efficiency of the oil extraction process. For three Italian cultivars, they evaluated the diffusion of oil and microconstituents determined by the disruption effects on olive cell tissues carried out by the non-thermal method. Their results show not only an increase in the percentage of oil extracted but also in the concentration of hydrophilic phenols.

All these contributions to OLIVEBIOTEQ'18 demonstrate the scientific community's synergism and cooperation with the industrial olive sector. Our interest is to provide knowledge and technology to study and solve the problems that the industry faces in the cultivation of olive trees, and in the production of high quality olive oil with the objective of increasing efficiency through the use of natural resources while ensuring the profitability for orchardists and producers. The increase in global population taken together with climate change pose new challenges to achieve that target, but this special issue confirms, once again, that science and technology are powerful tools to overcome new problems. The thanks of the committee behind the OLIVEBIOTEQ meetings go out to our colleagues for their continuous efforts to support olive growing and production.

## Author's Note

The authors were conveners of Olivebioteq'18, the 6th International Conference on Olive Tree and Olive Products, held at Seville in October 15-19, 2018.

## Author Contributions

All authors listed have made a substantial, direct and intellectual contribution to the work, and approved it for publication.

## Conflict of Interest

The authors declare that the research was conducted in the absence of any commercial or financial relationships that could be construed as a potential conflict of interest.

